# Claustrum Sign in Hashimoto Encephalopathy Presenting With New Onset Refractory Status Epilepticus (NORSE): First Report of a Rare Phenotype

**DOI:** 10.1002/ccr3.72382

**Published:** 2026-04-20

**Authors:** Zahra‐Sadat Mirian, Mohammad Amin Najafi, Alireza Anjam Majoumerd, Ali Khoshnevisan, Mohammad Reza Najafi

**Affiliations:** ^1^ School of Medicine Isfahan University of Medical Sciences Isfahan Iran; ^2^ Student Research Committee Isfahan University of Medical Sciences Isfahan Iran; ^3^ Department of Neurology Isfahan University of Medical Sciences Isfahan Iran; ^4^ Isfahan Neurosciences Research Center, Alzahra Hospital Isfahan University of Medical Sciences Isfahan Iran

**Keywords:** anti‐TPO autoantibodies, autoimmune encephalitis, claustrum sign, Hashimoto's encephalitis, NORSE, status epilepticus, steroid‐responsive encephalopathy associated with autoimmune thyroiditis

## Abstract

The presence of the bilateral claustrum sign in new‐onset refractory status epilepticus (NORSE) should prompt consideration of Hashimoto's encephalopathy, even without thyroid dysfunction. Early recognition and corticosteroid therapy can be lifesaving, emphasizing an autoimmune‐inflammatory mechanism underlying this rare presentation.

## 1 Introduction

New‐onset refractory status epilepticus (NORSE) is a rare condition characterized by seizures in a patient without active epilepsy or other preexisting neurological disorder (acute strokes, brain masses, drug overdoses, etc.), and without a clear acute or active structural, toxic, or metabolic cause, which is resistant to conventional seizure treatments. A consensus definition of NORSE and related conditions was established in 2018 to facilitate standardized terminology and improve patient care and clinical research [1]. The pathogenesis of NORSE has mainly remained vague. In a large retrospective review with 130 NORES cases, even after extensive evaluation, the etiology of 52% of cases remains unclear. These cases were termed as “cryptogenic NORSE” or “NORSE of unknown etiology.” The most common etiologies included autoimmune and paraneoplastic encephalitis, followed by infections [2].

Hashimoto's encephalopathy (HE) is one such autoimmune syndrome. It is associated with elevated antithyroid peroxidase (anti‐TPO) antibodies. The clinical presentation may involve a relapsing and remitting course and include seizures, stroke‐like episodes, cognitive decline, neuropsychiatric symptoms, and myoclonus [3]. Brain MRI findings in HE are often nonspecific and may appear normal in many cases [3, 4]. These changes are typically transient and may diminish with immunotherapy, making MRI a potentially helpful tool in early suspicion of autoimmune encephalopathy [3].

Presentation of HE with NORSE is quite rare, and even fewer reports describe status epilepticus as their initial presentation [4]. Here in we present the first ever reported case of NORSE in the setting of HE with the claustrum sign on brain MRI.

## 2 Case History/Examination

An 18‐year‐old male with no medical history was brought to our neurology center with status epilepticus, following a 10‐day history of worsening neurobehavioral symptoms. His neuropsychiatric symptoms began approximately 2 weeks after a brief episode of an upper respiratory tract infection, with low‐grade fever and coryza symptoms, which resolved on its own after 2 days.

His neuropsychiatric symptoms began gradually about 10 days before his arrival, including increasing irritability, social withdrawal, and reduced verbal output. His condition continued to worsen, with frequent focal seizures beginning 1 day before admission, described by his family as episodes of lip contraction and deviation to the right side, lasting 10–15 s. These occurred 4–5 times a day and eventually increased to nearly every hour, lasting up to 30 s. In parallel, his cognitive state progressively declined, though he remained awake but unconscious during the episodes.

He presented to our center with frequent focal seizures as described above. At the time of admission, he was alert but nonverbal, avoided eye contact, and exhibited inappropriate laughter along with persistent drooling. The seizures occurred approximately every hour, lasting 20–30 s, during which he remained conscious. Systemic examination revealed stable vital signs, afebrile, and there were no signs of meningeal irritation. Neurological assessment revealed mid‐sized, reactive pupils, no neck stiffness, symmetrical movement of all limbs, and bilateral down‐going plantar reflexes.

## 3 Differential Diagnosis, Investigations, and Treatment

Due to the progression of the patient's seizures and their transformation into status epilepticus, high‐dose antiepileptic therapy was initiated with levetiracetam 4 g stat, followed by 1.5 g three times daily. Despite treatment, the seizures progressed to generalized tonic–clonic convulsions. The patient was intubated, and midazolam was initiated (20 mg loading dose and 20 mg/h maintenance dose) along with a loading dose of sodium valproate (2400 mg), followed by 800 mg three times daily. He was subsequently transferred to the intensive care unit (ICU) for further management.

**FIGURE 1:**
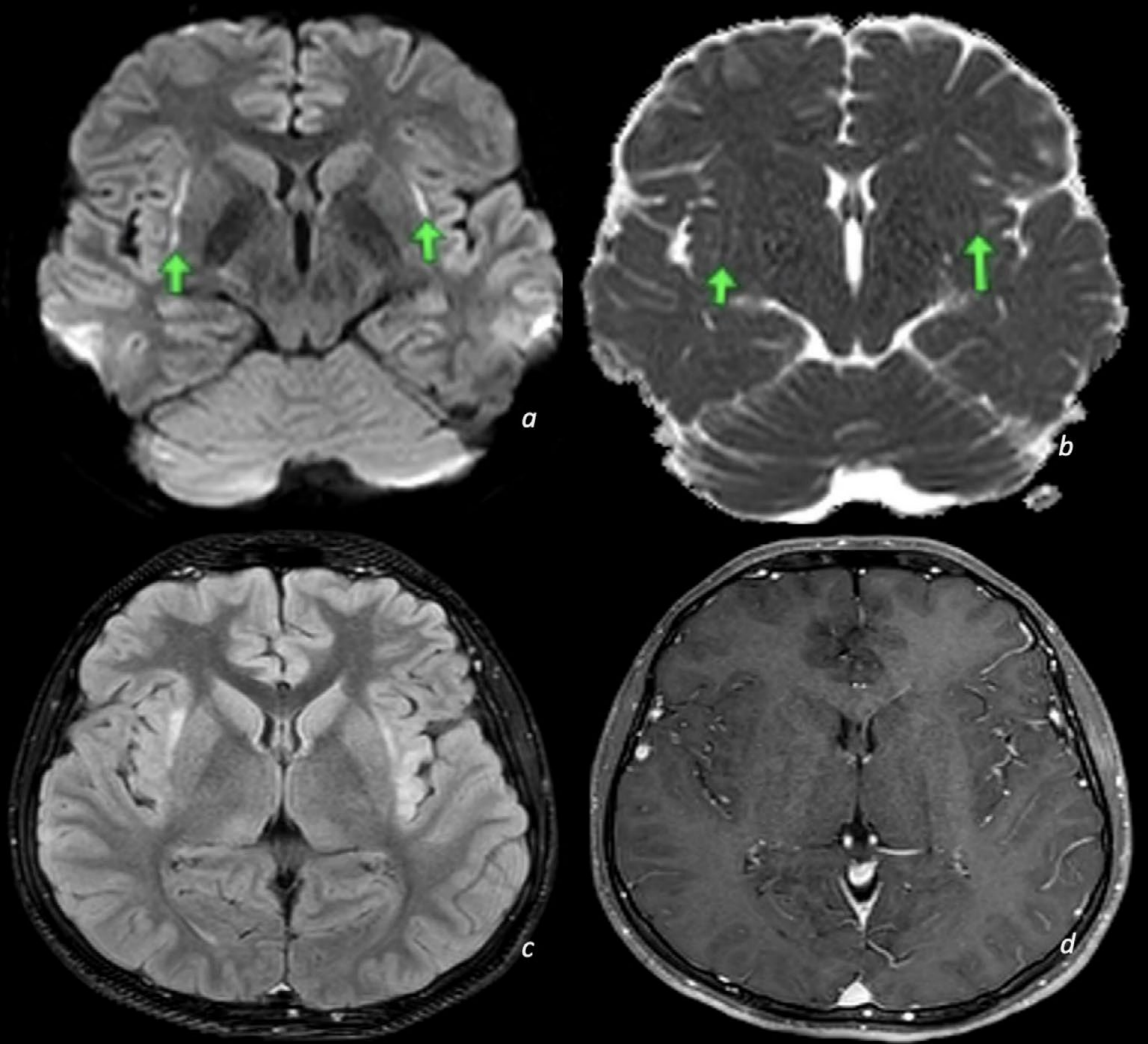
Axial diffusion‐weighted imaging (DWI) (a) and apparent diffusion coefficient (ADC) map (b) demonstrate bilateral restricted diffusion in the claustrum region. Axial FLAIR imaging (c) reveals hyperintensity in the bilateral claustrum and left insular cortices. Axial postcontrast T1‐weighted imaging (d) shows no contrast enhancement in the areas of signal abnormality identified on DWI and FLAIR.

A lumbar puncture was done, and analysis of the cerebrospinal fluid (CSF) was within normal range (no pleocytosis with three WBCs/mm^3^, a protein level of 26 mg/dL, and a glucose level of 38 mg/dL). An urgent brain MRI was performed and revealed bilateral claustrum hyperintensities and involvement of the left insular cortex (Figure 1).

The patient's clinical presentation was consistent with NORSE. The main differential diagnosis of NORSE includes autoimmune, paraneoplastic, and infectious etiologies. Autoimmune encephalitis associated with NORSE may show neuronal autoantibodies, including antibodies against the *N*‐methyl‐d‐aspartate (NMDA) receptor, Leucine‐rich, glioma inactivated 1 (LGI1), contactin‐associated protein‐like 2 (CASPR2), α‐amino‐3‐hydroxy‐5‐methyl‐4‐isoxazolepropionic acid (AMPA) receptor, Gamma‐Aminobutyric Acid_A (GABA_A)/GABA_B receptors, glycine receptor, and glutamic acid decarboxylase 65 (GAD65). Paraneoplastic cases most commonly involve anti‐NMDA receptor antibodies, antineuronal nuclear antibody‐type 1 (ANNA‐1), anticollapsin response mediator protein 5 (anti‐CRMP5), anti‐Ma2, antiamphiphysin, and antivoltage‐gated calcium channel (anti‐VGCC). Infectious etiologies are mainly caused by herpes simplex virus type 1 (HSV‐1), enterovirus, Epstein–Barr virus (EBV), varicella‐zoster virus (VZV), cytomegalovirus (CMV), *Bartonella henselae* , *Mycoplasma pneumoniae* , and arboviruses such as West Nile virus [2].

A comprehensive diagnostic workup was undertaken to assess the autoimmune, paraneoplastic, and infectious causes. Viral encephalitis was excluded by negative multiplex viral CSF PCR. Normal metabolic tests ruled out systemic causes. The bilateral claustrum sign suggested an autoimmune‐inflammatory process. Autoimmune and paraneoplastic panels through both serum and CSF were evaluated and were negative. Non‐neuronal antibodies, including antinuclear antibodies (ANA), antineutrophil cytoplasmic antibodies (ANCA), antiphospholipid antibodies, anticardiolipin, beta‐2 glycoprotein, lupus anticoagulant, rheumatoid factor (RF), double‐stranded DNA (dsDNA), and anti‐TPO, were all evaluated. Among these, anti‐TPO antibodies were found to be markedly elevated, exceeding 1000 IU/mL, which was rechecked and approved (Table 1).

**TABLE 1:** Laboratory findings (items marked with an asterisk (*) indicate significant findings).

Hormone			
T3*	90	ng/dL	91–218
T4	8.0	μg/dL	4.87–11.72
TSH	2.0	MIU/mL	0.3–5.1
IMMUNOLOGY			
Anti‐TPO*	> 1000	IU/mL	0–5.61

Based on highly elevated anti‐TPO antibodies, HE, also termed steroid‐responsive encephalopathy associated with autoimmune thyroiditis (SREAT), was the primary diagnosis. To further evaluate thyroid involvement, thyroid function tests and an ultrasound were performed. The thyroid ultrasound was unremarkable. And thyroid function tests were within normal limits. A malignancy workup was completed with additional whole‐body imaging (chest, abdominal, pelvic CT with and without contrast, and testicular sonography), which was negative.

**FIGURE 2:**
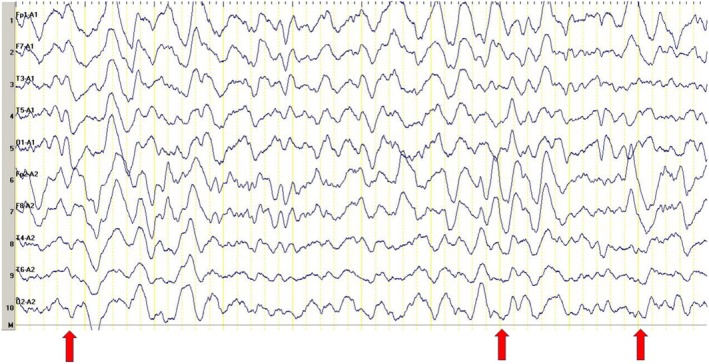
Scalp EEG recorded using a monopolar ipsilateral ear reference montage after 24 h reveals generalized sharp‐and‐slow wave and spike‐and‐slow wave discharges, further supporting the diagnosis of generalized seizure disorder. Sensitivity: 7 μV/mm, LFF: 1 Hz, HFF: 60 Hz.

**FIGURE 3:**
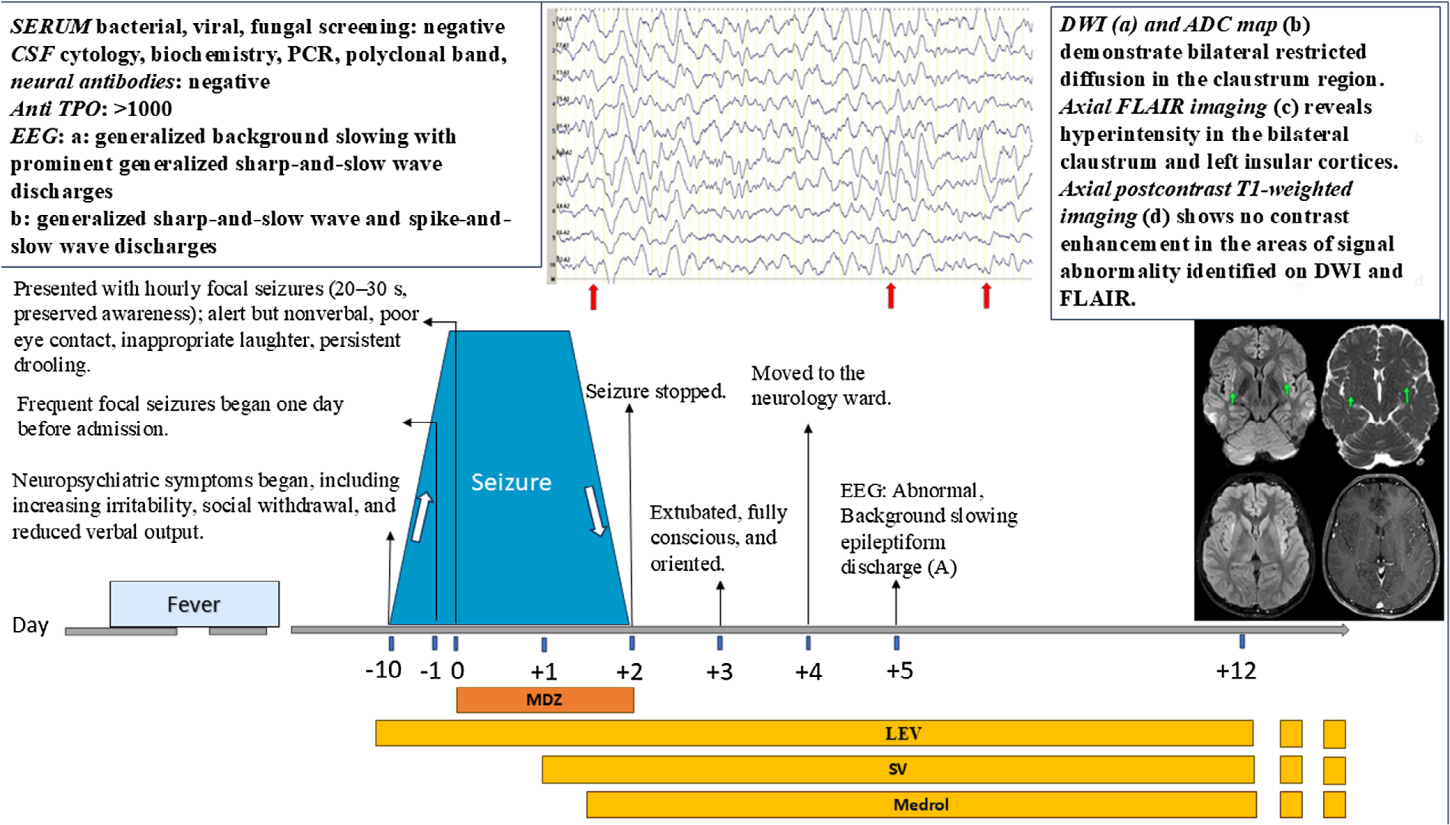
Patient's clinical course. CSF, cerebrospinal fluid; LEV, Levotiracciccam; MDZ, midazolam; SV, sodium valproate; Medrol, Methylprednisolone; MRI, Magnetic resonance imaging; DWI, Axial diffusion‐weighted imaging; ADC, apparent diffusion coefficient.

Based on these findings, we initiated high‐dose corticosteroid therapy with intravenous methylprednisolone (1 g daily for 5 consecutive days), in conjunction with empirical antimicrobial treatment. After 24 h, midazolam was discontinued, and the patient was seizure‐free. He was extubated after 48 h and, within the following day, transferred to the neurology ward in a fully conscious and stable condition. The EEG was abnormal, showing background slowing and generalized epileptiform discharges (Figure 2). After completing 5 days of high‐dose corticosteroids, oral prednisolone 1 mg/kg was started. During hospitalization, the patient became seizure‐free, cognitive symptoms improved, and the patient's mood symptoms improved significantly (Figure 3).

## 4 Discussion

Our patient was a previously healthy young male who presented with rapidly progressive neurobehavioral changes, seizure, and status epilepticus following an episode of an upper respiratory tract infection with low‐grade fever and coryza. The investigations revealed elevated anti‐TPO antibodies and bilateral claustrum sign in the Brain MRI. According to the patient's clinic, laboratory, and imaging findings, as well as a dramatic response to corticosteroid, the diagnosis of HE was established. This case is the first ever reported case of HE presented with NORSE and claustrum sign on brain MRI.

HE is a rare clinical condition associated with HT. Given its robust response to corticosteroid therapy, some experts introduced the term “SREAT” [5].

The pathogenesis of HE remains unclear. Still, proposed mechanisms include immune‐mediated cerebral vasculitis [5], an autoimmune response linked to antithyroid antibodies (particularly anti‐TPO) [6], and elevated central nervous system thyrotropin‐releasing hormone (TRH) levels [7]. While antithyroid antibodies support diagnosis, their levels do not correlate with disease severity [8].

Imaging findings in HE vary widely. In most cases, MRI scans are normal [9]; for instance, Castillo et al. reported that 74% of patients had either normal findings or nonspecific white matter involvement [10]. However, frequently reported abnormalities include bilateral FLAIR and T2 hyperintensities involving the mesial temporal lobes, caudate nuclei, and putamina, as well as a nonenhancing midbrain lesion, periventricular white matter hyperintensities, and meningeal enhancement [9, 11]. Less common findings, such as involvement of the splenium of the corpus callosum or the nucleus accumbens, have also been reported [12].

Some abnormalities have been shown to reverse completely with immunotherapy [13]. Brain MRI of our patient revealed the bilateral claustrum sign (typically described as symmetrical FLAIR or T2 hyperintensities in the claustrum region) and involvement of the insular cortex. While imaging findings in HE are highly variable and often nonspecific, the presence of bilateral claustrum hyperintensities has not been previously reported in this context. The claustrum sign is a rare but increasingly recognized radiologic feature in autoimmune or inflammatory encephalopathies. The claustrum sign has also been reported in other diseases such as Limbic encephalitis (LE), acute necrotizing encephalopathy (ANE), COVID‐19‐related encephalopathy, immune effector cell‐associated neurotoxicity syndrome (ICANS), and Wilson disease [14, 15, 16, 17].

Its appearance in our patient strengthens the presumed autoimmune‐inflammatory mechanism underlying both HE and NORSE, suggesting a shared pathophysiological pathway. This novel association highlights the importance of considering HE in the differential diagnosis when the claustrum sign is observed, particularly in the setting of NORSE.

## 5 Conclusion

We report the first documented case of HE presented as a NORSE, marked by the presence of the claustrum sign on brain MRI. This case highlights the importance of considering HE in the differential diagnosis of NORSE, particularly when the claustrum is involved radiologically. The dramatic response to immunotherapy further supports an autoimmune‐inflammatory etiology. Recognition of this rare presentation expands the phenotypic spectrum of HE. It reinforces the need for early immune‐targeted treatment in patients presenting with NORSE and autoimmune markers, even in the absence of overt thyroid dysfunction.

## Author Contributions

**Zahra‐Sadat Mirian:** conceptualization, investigation, visualization, writing – original draft, writing – review and editing. **Mohammad Amin Najafi:** conceptualization, investigation, methodology, project administration, supervision, visualization, writing – original draft, writing – review and editing. **Alireza Anjam Majoumerd:** conceptualization, visualization, writing – original draft, writing – review and editing. **Ali Khoshnevisan:** conceptualization, writing – original draft, writing – review and editing. **Mohammad Reza Najafi:** conceptualization, visualization, writing – original draft, writing – review and editing.

## Funding

The authors have nothing to report.

## Consent

Written informed consent was obtained from the patient to publish this case report.

## Conflicts of Interest

The authors declare no conflicts of interest.

## Data Availability

Data sharing is not applicable to this article as no datasets were generated or analyzed during the current study. All relevant clinical details are included within the manuscript.
